# Detection of Alzheimer’s Disease Using Logistic Regression and Clock Drawing Errors

**DOI:** 10.3390/brainsci13081139

**Published:** 2023-07-29

**Authors:** Sophia Lazarova, Denitsa Grigorova, Dessislava Petrova-Antonova, for the Alzheimer’s Disease Neuroimaging Initiative

**Affiliations:** 1 GATE Institute, Sofia University “St. Kliment Ohridski”, 1504 Sofia, Bulgaria; dessislava.petrova@gate-ai.eu; 2 Institute of Neurobiology, Bulgarian Academy of Sciences, 1113 Sofia, Bulgaria; 3 Faculty of Mathematics and Informatics, Sofia University “St. Kliment Ohridski”, 1504 Sofia, Bulgaria; dgrigorova@fmi.uni-sofia.bg

**Keywords:** Alzheimer’s disease, Alzheimer’s disease detection, clock drawing test, screening

## Abstract

Alzheimer’s disease is an incurable disorder that accounts for up to 70% of all dementia cases. While the prevalence of Alzheimer’s disease and other types of dementia has increased by more than 160% in the last 30 years, the rates of undetected cases remain critically high. The present work aims to address the underdetection of Alzheimer’s disease by proposing four logistic regression models that can be used as a foundation for community-based screening tools that do not require the participation of medical professionals. Our models make use of individual clock drawing errors as well as complementary patient data that is highly available and easily collectible. All models were controlled for age, education, and gender. The discriminative ability of the models was evaluated by area under the receiver operating characteristic curve (AUC), the Hosmer-Lemeshow test, and calibration plots were used to assess calibration. Finally, decision curve analysis was used to quantify clinical utility. We found that among 10 possible CDT errors, only 3 were informative for the detection of Alzheimer’s disease. Our base regression model, containing only control variables and clock drawing errors, produced an AUC of 0.825. The other three models were built as extensions of the base model with the step-wise addition of three groups of complementary data, namely cognitive features (semantic fluency score), genetic predisposition (family history of dementia), and cardio-vascular features (BMI, blood pressure). The addition of verbal fluency scores significantly improved the AUC compared to the base model (0.91 AUC). However, further additions did not make a notable difference in discriminatory power. All models showed good calibration. In terms of clinical utility, the derived models scored similarly and greatly outperformed the base model. Our results suggest that the combination of clock symmetry and clock time errors plus verbal fluency scores may be a suitable candidate for developing accessible screening tools for Alzheimer’s disease. However, future work should validate our findings in larger and more diverse datasets.

## 1. Introduction

Dementia is a devastating disease, causing gradual deterioration in cognitive function beyond the average age-related cognitive decline [1]. The disease is characterized by progressively worsening symptoms that slowly render patients unable to care for themselves, thus putting a tremendous physical, emotional, and financial burden on patients, caregivers, and society [2,3,4]. According to the World Health Organization (WHO), more than 55 million people worldwide are living with dementia, with about 10 million new cases every year [5]. Alzheimer’s disease is the most common form of dementia, corresponding to between 60 and 70% of all dementia cases [3]. While the prevalence of Alzheimer’s disease and other dementias has increased by 160.84% in the last 30 years [6], the disease remains underdiagnosed worldwide [4]. In 2017, researchers performed a systematic review of 23 articles reporting the proportion of undetected dementia in different parts of the world [7]. The authors found that the prevalence of undetected dementia in Asia was as high as 93.2%, followed by North America and Europe, with estimated rates of undetected dementia at 62.9% and 53.7%, respectively [7].

One of the phenomena leading to the mass underdiagnosis of dementia is the general lack of knowledge as well as the high prevalence of false beliefs about the disease. As a result, patients and caregivers often dangerously misinterpret dementia symptoms as a normal part of the aging process rather than an illness [8,9]. G.W. Ross reported that 21% of the informants of elderly Japanese-American men subsequently diagnosed with dementia failed to recognize memory deficiencies [10]. In 2004, P. Werner conducted in-depth interviews with 79 community-dwelling elderly persons and found that participants generally believed memory problems were an inevitable part of growing old and would seek help only if the problems became severe enough to disrupt their daily lives [11]. Similar beliefs were found in a sample of first-degree relatives of people with Alzheimer’s disease—19% of the participants assumed that significant memory loss was a normal part of aging [12]. It is important to note that community-level knowledge about Alzheimer’s disease appears to vary greatly across different countries. An online study conducted among Australian adults found that 88% of the respondents were able to correctly identify dementia from a vignette [13]. In contrast, the results from a similar study conducted in São Paulo, Brazil, showed that 46.4% of the participants identified AD symptoms as “memory loss”, while the term AD was used in only 4% of the responses. Furthermore, 39.4% of the respondents believed AD was a mental illness [14].

A possible solution to this problem is the introduction of community-based screening tests that can be administered outside of clinical settings and without the participation of medical professionals. Such automated screening will aid early detection of the disease and timely diagnosis by differentiating between individuals who probably have the disease and those who probably do not. However, to be suitable for community use, such a screening tool would have to use patient data that is informative and available for collection by anyone. While there is a great variety of newly developed screening methods and biomarkers, a large portion of them are limited because they require a controlled environment. Some examples are blood-based biomarkers [15,16,17,18], ophthalmologic biomarkers based on eye movement data [19], retinal texture [20], and vascular condition of the retina [21], and combined models leveraging multifaceted patient data that can have significant levels of complexity [22,23,24,25]. Some alternative approaches offer greater levels of flexibility and availability. For instance, screening based on speech patterns [26,27] and mobile applications [28,29] leverages digital versions of widely used cognitive tests.

The Clock Drawing Test (CDT) was originally introduced in the early 20th century as a test for constructional apraxia, used to assess visuoconstructional disorders associated with lesions in the parietal lobe [30]. The relevance of the CDT for discrimination between AD and healthy elderly individuals has been demonstrated in the context of the traditional paper-based test [30,31,32] as well as in digitalized versions of the test [33,34], some of which take into account not only the final score but the whole process of drawing [35]. Furthermore, previous studies showed that CDT successfully discriminates between AD pathology groups in cognitively healthy older adults [36]. The test consists of asking the subject to draw the face of a clock with the clock hands set at a particular time. In some variations, the CDT also contains a "copy" condition where the subject is also asked to copy a clock from a given example. A considerable benefit of CDT is its simplicity, due to which it can be easily administered by non-professionals such as family members, friends, or caregivers. Furthermore, the CDT can be easily digitalized and even automated.

While the total CDT score is traditionally used for patient evaluation, several studies have demonstrated the value of analyzing individual clock drawing errors in tasks related to dementia detection. For example, Lessig et al. found that among 24 clock drawing errors, a subset, as small as six errors, can be used to identify dementia with 88% specificity and 77% sensitivity [37]. Other studies examined the quantitative and qualitative CDT errors committed in Azheimer’s disease, Parkinson’s disease, and Huntingtun’s disease [38,39,40]. The results of these studies demonstrate that certain clock errors can be used to differentiate between the neuropsychological profiles of these diseases, suggesting that individual clock errors can be incorporated as part of screening models and models aiding differential diagnosis. The analysis of individual clock errors also appears helpful in staging AD. Previous work has demonstrated that the severity of the disease is associated with the increased presence of specific mistakes [37,38]. Similarly, Suzuki et al. found that the increased number of clock errors caused by conceptual deficits is associated with a higher risk of falling in AD patients [41].

The present work is motivated by the need for more models suitable for first-line community-based AD screening in non-medical settings and without the participation of medical professionals. We offer variations of logistic regression models for the detection of AD based on individual clock drawing errors and complimentary patient data collected within several minutes. To guarantee maximum simplicity and usability, the requirements for the data included in the here-presented models can be summarized as follows:

- All necessary data should be collected within a few minutes;
- The collection of the data should not require a medical professional;
- Necessary data should be simple and thus suitable for comprehension by the general population.

The rest of the paper is organized as follows: Section 2 describes the data and the methods used within this work; Section 3 reports the obtained results; and Section 4 offers a discussion of the results by putting them in the context of previous findings. Finally, Section 5 examines the limitations of the present work as well as future directions and developments.

## 2. Materials and Methods

### 2.1. Study Design and Aims

The primary objective of this study was to evaluate the predictive capability of CDT errors in detecting amnestic Alzheimer’s disease. The study analyzed errors made by controls and AD patients in both “command” and “copy” conditions of the CDT and their significance in predicting Alzheimer’s disease status using a logistic regression model. Additionally, the study investigated the possibility of enhancing the predictive capacity of the CDT-based model by supplementing it with relevant data such as verbal fluency scores, body mass index (BMI), blood pressure (BP), and family history of dementia.

### 2.2. Data Source

The data used in the present study were obtained from the Alzheimer’s Disease Neuroimaging Initiative (ADNI) database. ADNI is an ongoing longitudinal study launched in 2004 that aims to understand the changes occurring during the progression of Alzheimer’s disease. Full details and protocols can be found on the ADNI website [42].

### 2.3. Data Selection

The ADNI study follows volunteers with a baseline age between 55 and 90 years, each assigned to one of the following groups: cognitively normal (CN), mild cognitive impairment (MCI), or Alzheimer’s disease (AD). In the present study, we focused only on the CN and AD groups. The participant inclusion criteria for these groups are available in Table 1. Further details are available in the official ADNI procedure manual [43].

As seen in Table 1, the ADNI group inclusion criteria heavily depend on Mini-Mental State Exam scores as well as the Wechsler Memory Scale. While the Mini-Mental State Exam is a commonly used tool for fast assessment of mental state, the Wechsler Memory Scale is a neuropsychological scale designed to test several memory functions—auditory memory, visual memory, visual working memory, immediate memory, and delayed memory. Therefore, utilizing any of these variables or comparable ones as predictive factors could potentially produce substantially biased outcomes; consequently, our options for complimentary cognitive measures were restricted. Therefore, the current investigation includes only the verbal fluency score as an additional factor from the cognitive domain, in addition to the Clock Drawing Test (CDT). Tasks of verbal fluency involve both language and executive functions and are consistently found to be impaired in AD populations in comparison with a normative group [44,45]. Therefore, verbal fluency complements the CDT as an additional measure of executive function. According to the work of Weakly and Schmitter-Edgecombe, executive abilities involving search and retrieval processes and a reduced availability of semantically related words are contributing to the poor performance of AD populations on verbal fluency tasks [44]. Moreover, verbal fluency can be predictive of the incidence of cognitive impairment, as demonstrated by Sutin et al. In their work, they found that every standard deviation increase in verbal fluency was associated with an approximately 60% reduced risk of incident dementia [45]. Finally, verbal fluency tasks are simple and can be performed anywhere. In fact, Kwon et al. demonstrated the utility of verbal fluency tasks as a self-administered screening tool by proposing a semi-automated Android app that achieved an AUC of 0.86 for AD detection [29].

Similarly, body mass index, blood pressure, and family history of dementia are easily collectible and have been associated with the development of the disease. While a higher BMI is considered a risk factor for the development of Alzheimer’s disease, gradual weight loss is expected near the onset and past the beginning of the disease due to cardiometabolic changes [46]. Hypertension has also been associated with AD; in particular, high BP has been found to modulate the relationship between cerebral Aβ and tau deposition [47].

### 2.4. Data Description

We obtained baseline records for 943 ADNI participants classified as either cognitively normal (CN) or Alzheimer’s disease (AD). We included anamnestic data, results from a clock drawing task, a verbal fluency task, and height, weight, and blood pressure measurements for each participant. The anamnestic data included age, gender, years of education, and family history of dementia. Family history of dementia was defined as a binary variable denoting the presence (or absence) of diagnosed parental dementia. Similarly, the gender of each participant was represented by a binary variable, where 1 signifies a female. Additionally, we calculated BMI derived from height and weight.

**Figure 1:**
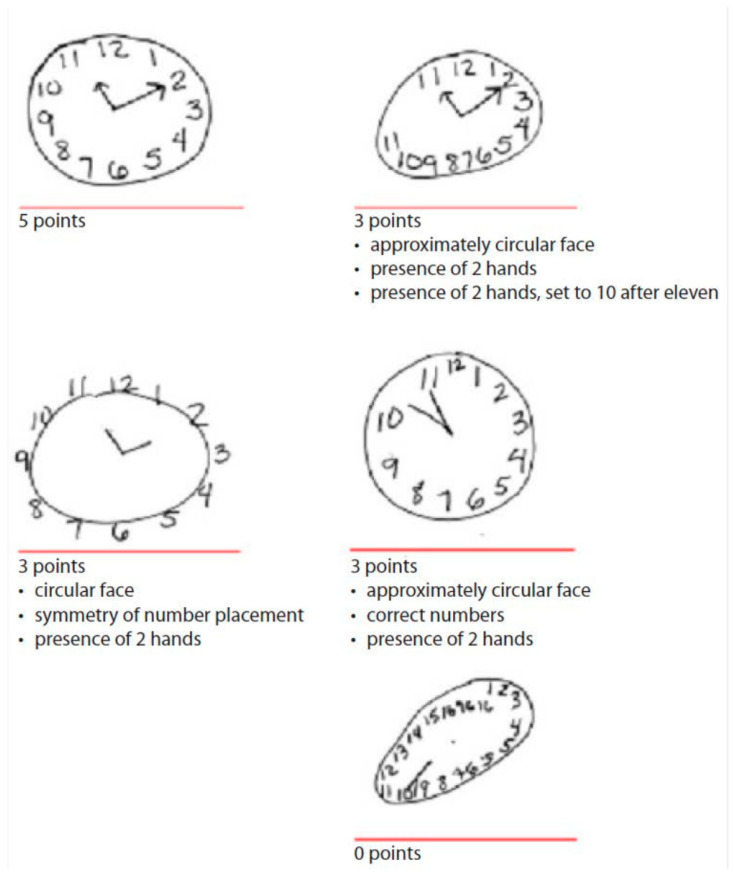
Scoring clock drawings. The image was copied from the ADNI 3 Procedures Manual [43].

The clock drawing task consisted of two components: a command condition in which the subject was asked to draw a clock according to verbal instructions and a copy condition in which the subject was asked to copy a sample clock drawn at the top of the response form. Clock drawings were scored on a scale from 0 to 5, with 5 corresponding to the best performance (Figure 1). Each drawing was given 1 point for meeting each of the following criteria:

1. Drawing approximately a circular face
2. Placing clock numbers symmetrically
3. Correct clock numbers (must have all numbers in the correct order, placed inside the circle)
4. Presence of two hands
5. Presence of two hands set to ten after eleven

Therefore, for each ADNI participant, we obtained a total of 10 binary CDT variables—5 corresponding to the command condition (Draw circle, clock symmetry, clock numbers, clock hands, clock time) and 5 representing the performance on the copy condition (Copy circle, copy symmetry, copy numbers, copy hands, copy time).

### 2.5. Data Analysis

All statistical analyses were conducted using RStudio version 2022.07.2 + 576 “Spotted Wakerobin” Release and R version 4.2.1 [48]. As appropriate, comparisons between the AD and CN groups were performed using a one-way ANOVA test or the chi-squared test of independence. *p*-values less than 0.05 were considered statistically significant results. The individual association between each feature and AD diagnosis was estimated with odds ratios and 95% confidence intervals in age-, education-, and gender-adjusted logistic regressions.

#### 2.5.1. Model Development

We leveraged logistic regression models to examine the capability of clock drawing errors to discriminate between individuals with Alzheimer’s disease and those without the condition. Logistic regression is a classical statistical method for modeling binary outcomes that is commonly preferred for medical applications due to its high level of interpretability. Although machine learning methods often produce superior results on high-dimensional data, their complexity makes them more challenging to understand and interpret. Furthermore, in low-dimensional data, machine learning methods perform similarly to logistic regression [49]. Given that our data possesses low-dimensional characteristics and the advantage of interpretability provided by logistic regression, we opted for logistic regression over machine learning techniques.

The present work compares four regression models for AD detection: a base model, considering only the CDT errors as predictive factors, and three derived models, each extending the base model with a complementary group of predictors. All models were controlled for age, gender, and education to account for the influence of normal age-related aging and education on cognitive function as well as any gender-mediated differences between the groups.

The base model included the full range of CDT errors as predictors and the diagnosis as a dependent variable. Note that two CDT errors, namely “copy circle” and “copy hands”, were not included in the analyses due to low rates of errors among the participants. A backward elimination procedure was employed to exclude non-significant features and optimize the model. A standard 5% significance level was used to perform a step-wise exclusion of predictors with *p*-values > 0.05, starting from the predictor with the highest *p*-value.

Three derived models were built by extending the base model with three groups of relevant health data: cognitive features (verbal fluency score), genetic predisposition (family history of dementia), and cardio-vascular features (BMI, blood pressure). Each group was added to the model on a one-by-one basis, followed by a backward elimination step.

#### 2.5.2. Model Evaluation

We assessed the performance of the models in three domains: discrimination, calibration, and the benefits of clinical use. Discrimination was evaluated with the area under the receiver operating characteristic curve (ROC), also known as the area under the curve (AUC). AUC values and ROC can be used to evaluate the diagnostic ability of tests to discriminate the true state of subjects [50]. Thus, they are extensively used in clinical epidemiology to assess the diagnostic ability of biomarkers in classifying diseased and healthy individuals [50]. ROC curves and AUC were derived with functions from the pROC package [51]. Differences between the obtained ROC curves were examined with Delong’s test for correlated ROC curves [52].

Calibration curves and the Hosmer-Lemeshow test were plotted for each model to examine the concordance between the produced and observed probabilities of AD diagnosis. A significant result (*p*-value < 0.05) on the Hosmer-Lemeshow test denotes that the model does not calibrate well. Calibration was evaluated with the modEva package [53]. While calibration performance is often overlooked, it is essential for decision-support models [54], and the importance of reporting it has also been emphasized by the TRIPOD (Transparent Reporting of a Multivariable Prediction Model for Individual Prognosis or Diagnosis) guidelines for prediction modeling studies [55]. Finally, we used decision curve analysis to evaluate the benefits of clinical use [56].

## 3. Results

### 3.1. Sample Characteristics

Out of 943 participants, 47 were excluded due to missing data, leading to a final sample of 896 participants (mean age 74 ± 7.07 years; 51% male)—384 AD and 512 CN participants.

ANOVA tests showed that the individuals in the AD group were slightly older and less educated (Table 2). As expected, the AD group was characterized by lower BMI levels and exhibited considerably inferior results on the verbal fluency task compared to the CN group. However, the two groups did not differ in terms of mean systolic and diastolic blood pressure (Table 2).

A series of chi-square tests showed that the AD group had a significantly higher proportion of males in comparison to the CN group. Still, the two groups did not differ regarding family histories of dementia (Table 3). In terms of CDT performance, as expected, the CN group performed consistently better and had lower rates of error on all components compared to the AD group (Table 3). While there is a clear statistical dependence between all types of CDT errors and AD diagnosis, it is important to note that the results from the chi-square tests for the CDT components "copy circle" and "copy hands" were treated as unreliable since the error rates on these two components were extremely low among the CN group (Table 3).

### 3.2. Logistic Regression Models for the Detection of AD

The base model contained three significant CDT components, namely clock symmetry, clock hands, and clock time. Successfully executing the clock symmetry component decreased the odds of having AD three times (OR 0.33, 95% CI [0.22, 0.49]). Similarly, drawing a clock that meets the clock hands (OR 0.27, 95% CI [0.11, 0.67]) and clock time (OR 0.15, 95% CI [0.11, 0.22]) criteria lowered the odds of having AD 3.8 and 6.5 times, respectively. Additional years of education (OR 0.89, 95% CI [0.83, 0.94]) and being female (OR 0.54, 95% CI [0.39, 0.76]) were also found to decrease the odds of having AD.

**Figure 2:**
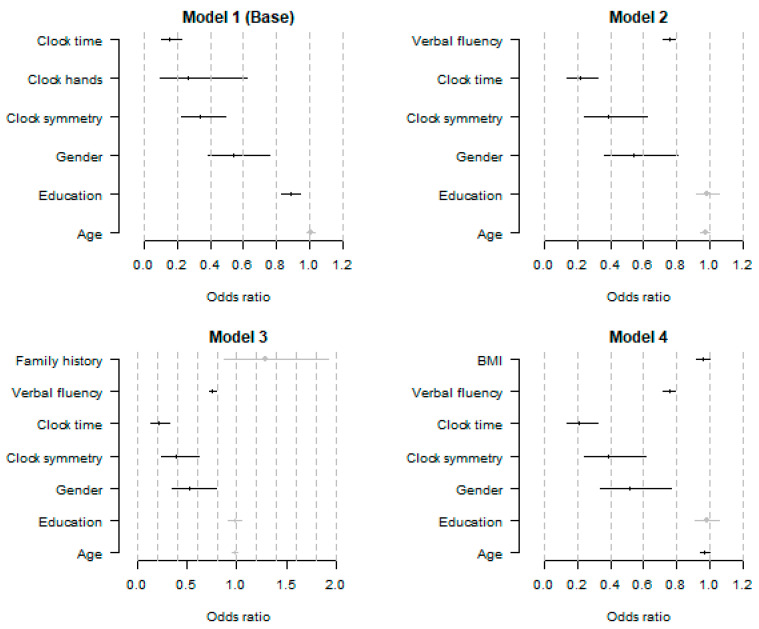
Odds ratios of the variables in each model—point estimate and 95% confidence interval. Grey points designate non-significant variables.

Adding verbal fluency to the base model led to the exclusion of clock hands due to statistical insignificance (Table 4, Model 2). Each additional correct word on the verbal fluency task decreased the odds of having AD 1.33 times (OR 0.75, 95% CI [0.72, 0.79]). In contrast, a family history of dementia did not affect the odds of AD; thus, adding it to the second model did not lead to any significant changes (Table 4, Model 3). Last, model 4 showed that a higher one-unit BMI slightly decreases the odds of having AD (OR 0.96, 95% CI [0.92, 0.99]). Model 4 was obtained by adding BMI and BP to Model 3. However, both systolic and diastolic blood pressures were excluded during the backward elimination step (Table 4, Model 4). All odds ratios and results from the logistic regressions are presented in Figure 2 and Table 4. Regarding control variables, only the female gender was consistently associated with lower odds of having AD across all models.

### 3.3. Discrimination of Logistic Regression Models for AD Detection

**Figure 3:**
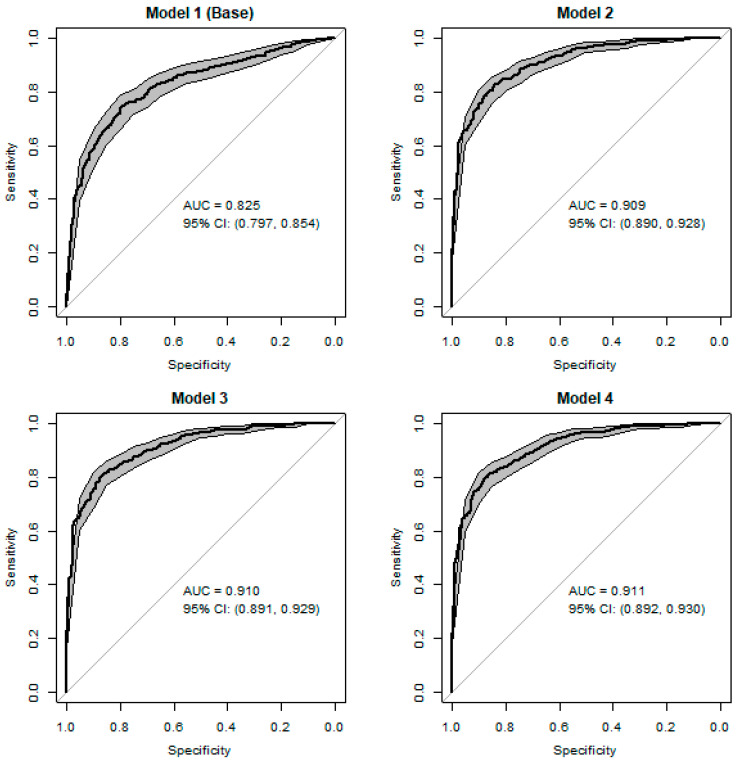
Area under the curve with 95% confidence intervals. Model 1 (base) = * + clock hands; Model 2 = * + verbal fluency; Model 3 = * + verbal fluency + family history of dementia; Model 4 = * + verbal fluency + BMI, where * designates the following five variables: age, education, gender, clock symmetry, and clock time.

In terms of discrimination, all models showed satisfactory performance, with the base model showing decreased discriminatory capability compared to the derived models (Figure 3). While the addition of verbal fluency led to a substantial improvement in AUC (Base Model—AUC 0.825 [95% CI 0.797–0.854]; Model 2—AUC 0.909 [95% CI 0.797–0.854]), none of the further added variables led to any noticeable improvements in terms of discrimination AUC (Model 3—AUC 0.910 [95% CI 0.891–0.929]; Model 4—AUC 0.911 [95% CI 0.892–0.930]). Delong’s test for correlated ROC curves showed that while all of the derived models demonstrated significantly better performance compared to the base model (Base vs. Model 2: Z = −7.1, *p*-value < 0.0001; Base vs. Model 3: Z = −7.1, *p*-value < 0.0001; Base vs. Model 4: Z = −7.2, *p*-value < 0.0001), there was no statistical difference between the AUCs obtained from the derived models. Therefore, the results suggest that in terms of discriminatory power, Model 2 has significant advantages over the other models, namely a high AUC (0.91) and a minimal set of predictors (compared to Models 3 and 4).

### 3.4. Calibration of Logistic Regression Models for AD Detection

**Figure 4:**
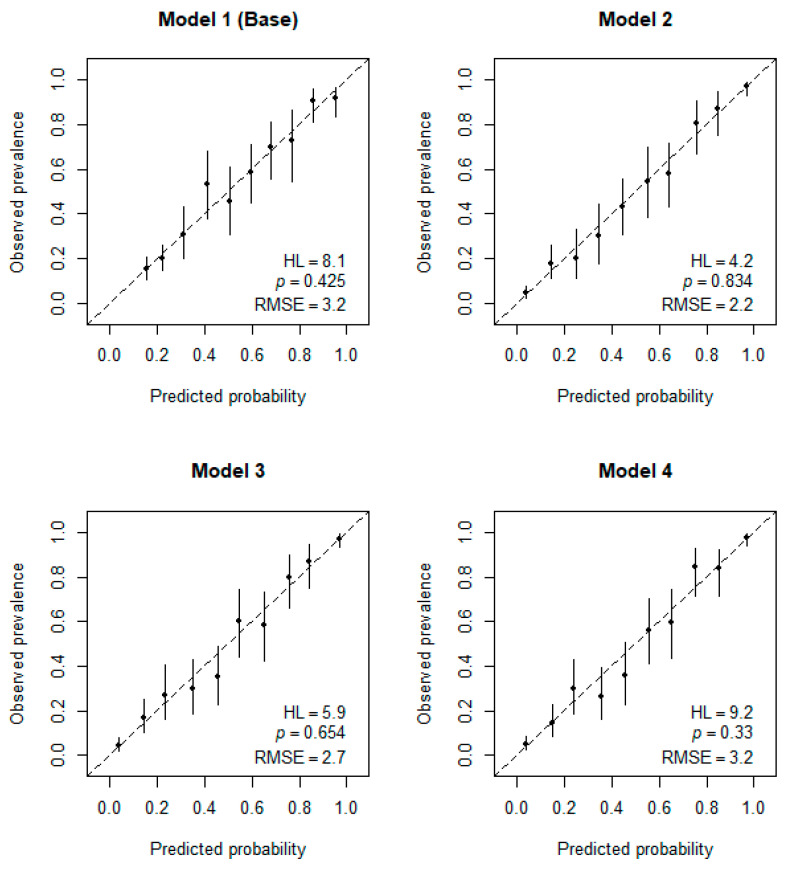
Calibration curves and HL-test results. *p* > 0.05 indicates good agreement between predicted and observed AD incidence.

According to the Hosmer and Lemeshow test, all models show good agreement between the predicted probability and the observed incidence of AD (Figure 4, *p*  >  0.05). However, model 2 demonstrated the highest level of agreement (HL = 4.2, *p* = 0.834), thus showing that family history of dementia and BMI do not introduce any improvements in terms of calibration.

### 3.5. Clinical Utility of Logistic Regression Models for AD Detection

**Figure 5:**
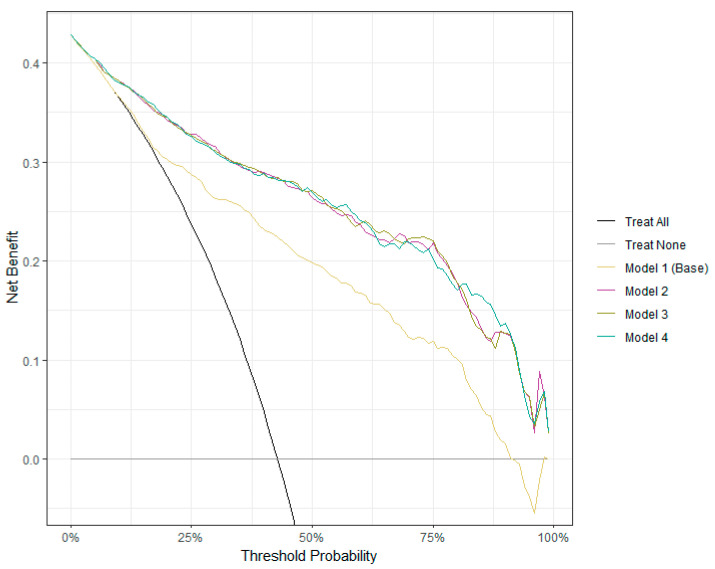
Decision curve analysis (DCA) results. The x-axis shows the continuum of potential thresholds for AD risk, and the y-axis shows the net benefit of using the models to stratify patients according to risk. "Treat all" refers to the assumption that all patients are assumed to probably have AD, and "Treat none" refers to the assumption that all patients are assumed to probably not have AD.

Decision curve analysis showed that all models have higher net benefits compared to the default—“treat all” and “treat none” (Figure 5). However, the base model scores lower on the entire range of threshold probabilities than the derived models. While in the lowest range of threshold probability, the derived models produce a net benefit very similar to the one made by the “treat all” strategy, the derived models outperform all other alternatives between 25% and 100%. Thus, we can conclude that intervening with patients based on the results of any of the derived models leads to higher benefits than the alternative strategies of “treat all," “treat none," and the base model. In the context of our work, “treat all” and “treat none” strategies refer to examining all patients as if they have AD and considering that all patients do not have it. Since our models are conceived as screening models, we would expect them to point correctly to patients at risk of AD while minimizing unnecessary examinations.

## 4. Discussion

In the present study, we demonstrated the usability of the individual clock drawing test errors for predicting Alzheimer’s disease. First, we built a logistic regression model including the CDT errors and controlling for age, education, and gender. Then, using backward elimination, we obtained a base model (Model 1), which featured three CDT errors as predictors and achieved a 0.83 AUC. Next, we extended the base model with several groups of additional variables to examine whether its performance could be improved. We demonstrated that the base model can be significantly improved by adding a single variable: verbal fluency score (0.91 AUC, Model 2). However, further additions (family history, BMI, blood pressure) did not improve the model’s performance (Models 3 and 4). Similarly, Model 2 had the highest concordance between predicted and observed probabilities of AD. In terms of clinical utility, all derived models demonstrated comparable net benefits, outperforming all alternative strategies. Thus, our results suggest that the combination of clock symmetry and clock time errors plus verbal fluency scores may be a suitable candidate for developing accessible screening tools for Alzheimer’s disease.

The generally accepted interpretation of AUC considers values of AUC around 0.5 as showing no discriminatory power; AUC between 0.7 and 0.8 is considered acceptable; AUC between 0.8 and 0.9 is excellent; and finally, AUC greater than 0.9 is considered outstanding [57]. Thus, Model 1 falls within the "excellent" band, and the rest of the models achieve AUCs that classify them as "outstanding" regarding their discriminatory power. Thus, we consider the obtained results positive and encouraging in demonstrating the suitability of the CDT as a foundation for community-based screening tests. Nevertheless, it should be noted that while our models show excellent results on the ADNI dataset, we cannot guarantee that the same models will perform equally well on other datasets. Further research is needed to conclude that the set of features used in the present work represents actual differences in the population rather than sample characteristics.

While our results support previous findings demonstrating the application of the CDT for dementia screening, they also emphasize the importance of individual CDT errors about the state of AD. As a result of excluding all non-significant predictors, our initial model used only three CDT errors as predictors: clock symmetry, clock hands, and clock time. The fact that not all errors appear to relate significantly to the diagnosis of Alzheimer’s disease strongly suggests that considering individual errors instead of compound scores might be a better strategy in the context of dementia classification. The significance of the clock symmetry, clock hands, and clock time errors is in line with previous studies presenting qualitative error analysis on clock drawings in AD patients. The results of two similar studies showed that the most common errors in AD patients were related to conceptual, spatial, and planning deficits [38,39]. Both clock hands and clock time reflect conceptual deficits as they refer to some misconceptions about the clock, particularly about the position, appearance, and presence of the hands and the appropriate representation of the time [39]. Similarly, clock symmetry errors manifest spatial and/or planning deficits since they refer to deficiencies in the layout of the clock, in particular the symmetry of the numbers [39]. Thus, the Clock Drawing Test (CDT) is a rapid, affordable, easy-to-administer, non-invasive test that may have some advantages as an early screening tool for detecting Alzheimer’s disease over other more expensive tests that cannot be administered outside the clinical setting.

## 5. Limitations & Future Directions

While we consider the presented results encouraging, we acknowledge several limitations to our work. First, the significance of the predictors used is relevant only to the data on which the feature selection was performed. Since our models were built on ADNI data and were not tested on any other data set, we refrain from making any claims regarding the significance of the models outside of the used data. To confirm the general validity of our findings, future work should test the validity of the models on different and preferably larger datasets.

Although the NINCDS/ADRDA diagnostic criteria used by the ADNI are still clinically relevant, they have undergone major revisions over the years due to the evolving understanding of the disease. As a result, this work considers only the amnestic variant of Alzheimer’s disease and does not take into account other variants characterized by other symptoms than memory impairment. To be in line with the latest advancements in the field, future work will focus on comparing healthy subjects with individuals diagnosed with histopathologically defined Alzheimer’s disease. Similarly, assessing the predictive value of the clock test in other variants of Alzheimer’s disease is a topic that also remains to be addressed in the future.

On a similar note, the present work considers only verbal fluency as a complementary measure of cognitive function. However, other dimensions of cognitive function such as visuospatial, executive, attentional, and abstractive functions might also prove useful in the detection of Alzhemer’s disease based on clock drawing errors.

Another limitation of our work is that only one scoring system of clock drawings was considered due to data availability limitations. Since ADNI offers only scores from the CDT and not actual drawings, we were limited in our choice of scoring systems. Nevertheless, this limitation does not render our results less meaningful since most scoring systems have been validated in their ability to distinguish between healthy elderly adults and adults with dementia [58]. However, more complex scoring systems with a higher level of error granularity may produce superior results, especially when considering MCI or preclinical cohorts.

Finally, it is essential to note that our study might be susceptible to sampling bias due to using ADNI. ADNI cohorts consist of volunteers included in the studies, provided they meet a particular set of inclusion criteria. Thus, at minimum, ADNI studies are prone to self-selection bias and might not be fully representative of the population.

Future work should focus on validating the presented results with external data sets that are ideally larger and more varied. Furthermore, such work should address the full spectrum of the disease, including individuals with histopathologically defined Alzheimer’s disease and variants other than the amnestic form of the disease. The successful implementation of community-based screening tests requires reliable automation of data collection as well as automated evaluation of drawings. Thus, future work should focus on building a digital version of the CDT that incorporates automated scoring of drawings based either on computer vision and deep learning or predefined scoring systems. Currently, such work is limited, mainly due to the need for labeled datasets containing the original clock drawings. Therefore, the scientific community would greatly benefit from efforts to collect such data and openly provide it for scientific purposes.

## Figures and Tables

**Table 1:** Summarized representation of the inclusion criteria used in the ADNI studies.

ADNI Group Inclusion Criteria	
CN Group	AD Group
No memory complaints aside from those common to other normal subjects of that age range.	Reported memory complaint by patient or study partner
Normal memory function score on the Wechsler Memory Scale (adjusted for education)	Abnormal memory function score on the Wechsler Memory Scale (adjusted for education)
Mini-Mental State Exam score between 24 and 30 (inclusive)	Mini-Mental State Exam score between 20 and 26
Clinical Dementia Rating = 0; Memory Box score must be 0	Clinical Dementia Rating = 0.5; Memory Box score at least 1.0
Cognitively normal, based on the absence of significant impairment in cognitive functions or activities of daily living.	NINCDS/ADRDA criteria for probable AD

**Table 2:** Descriptive statistics (mean ± sd) of the numerical variables. Group means were compared with ANOVA F tests. Significance levels are designated as follows: *** < 0.001; ** < 0.01, * < 0.05.

Variable	AD	CN	*p*-Value (ANOVA F Test)
Age	74.78 ± 7.94	73.44 ± 6.28	0.0049 **
Education (in years)	15.17 ± 2.92	16.42 ± 2.59	<0.0001 ***
BMI	25.96 ± 4.75	27.32 ± 8.27	0.0040 **
Systolic BP	133.80 ± 17.45	132.80 ± 16.27	0.3913
Diastolic BP	73.80 ± 9.65	73.90 ± 9.80	0.8835
Verbal fluency	12.21 ± 5.07	21.15 ± 5.62	<0.0001 ***

**Table 3:** Descriptive statistics of gender, family history of dementia, and Clock Drawing Test (CDT) components. The percentages in parentheses represent the conditional distribution of failure and success given the diagnosis. The dependence between each variable and the diagnosis was assessed with a Chi-square test. Shaded rows represent CDT components with critically low rates of error. Significance levels are designated as *** < 0.001, ** < 0.01, * < 0.05.

Variable	AD		CN		*p*-Value (Chi-Square Test)
Gender	Male	Female	Male	Female	0.0060 **
	218 (56.77%)	166 (43.23%)	242 (47.27%)	270 (52.73%)	
Family history	No	Yes	No	Yes	0.3493
	205 (53.39%)	179 (46.61%)	256	256	
			(50%)	(50%)	
**Clock Component**	**AD**		**CN**		***p*-Value (Chi-Square Test)**
	Failure (0)	Success (1)	Failure (0)	Success (1)	
Clock circle	13	371	4	508 (99.22%)	0.0099 ***
	(3.39%)	(96.61%)	(0.78%)		
Clock symmetry	177 (46.09%)	207	61	451 (88.09%)	<0.0001 ***
		(53.91%)	(11.91%)		
Clock numbers	114 (29.69%)	270	36	476 (92.97%)	<0.0001 ***
		(70.31%)	(7.03%)		
Clock hands	79	305	6	506 (98.83%)	<0.0001 ***
	(20.57%)	(79.43%)	(1.17%)		
Clock time	240	144	68	444 (86.72%)	<0.0001 ***
	(62.5%)	(37.5%)	(13.28%)		
Copy circle	7	377	1	511	0.0275 *
	(1.82%)	(98.18%)	(0.2%)	(99.8%)	
Copy symmetry	95	289	33	479 (93.55%)	<0.0001 ***
	(24.74%)	(75.26%)	(6.45%)		
Copy numbers	42	342	6	506 (98.83%)	<0.0001 ***
	(10.94%)	(89.06%)	(1.17%)		
Copy hands	30	354	0	512	<0.0001 ***
	(7.81%)	(92.19%)	(0%)	(100%)	
Copy time	107 (27.86%)	277	30	482 (94.14%)	<0.0001 ***
		(72.14%)	(5.86%)		

**Table 4:** Results from the model fit. Control variables are marked in bold (age, education, and gender). The reference levels for the categorical variables are gender (male), family history of dementia (no), and clock drawing test errors (failure). The number of stars designates the significance level: *** < 0.001, ** < 0.01, * < 0.05. Only predictors that survived backward elimination are listed.

Predictor	Estimate	Standard Error	Z-Test	*p*-Value
Model 1 (Base model)				
Intercept	4.580	1.149	3.99	<0.0001 ***
**Age**	**0.008**	**0.012**	**0.69**	**0.492**
**Education**	**−0.121**	**0.031**	**−3.85**	**0.0001** ***
**Gender (female)**	**−0.613**	**0.174**	**−3.53**	**0.0004** ***
Clock symmetry (success)	−1.094	0.199	−5.49	<0.0001 ***
Clock hands (success)	−1.328	0.470	−2.83	0.0047 **
Clock time (success)	−1.868	0.186	−10.02	<0.0001 ***
Model 2				
Intercept	8.558	1.342	6.38	<0.0001 ***
**Age**	**−0.026**	**0.014**	**−1.80**	**0.072**
**Education**	**−0.015**	**0.036**	**−0.42**	**0.673**
**Gender (female)**	**−0.613**	**0.205**	**−2.99**	**0.0028** **
Clock symmetry (success)	−0.947	0.239	−3.97	<0.0001 ***
Clock time (success)	−1.541	0.214	−7.19	<0.0001 ***
Verbal fluency (success)	−0.282	0.023	−12.34	<0.0001 ***
Model 3				
Intercept	8.303	1.355	6.13	<0.0001 ***
**Age**	**−0.022**	**0.014**	**−1.54**	**0.123**
**Education**	**−0.020**	**0.037**	**−0.54**	**0.586**
**Gender (female)**	**−0.638**	**0.207**	**−3.09**	**0.002** **
Clock symmetry (success)	−0.959	0.239	−4.01	<0.0001 ***
Clock time (success)	−1.549	0.215	−7.20	<0.0001 ***
Verbal fluency (success)	−0.283	0.023	−12.33	<0.0001 ***
Family history (yes)	0.255	0.202	1.26	0.206
Model 4				
Intercept	10.050	1.542	6.52	<0.0001 ***
**Age**	**−0.029**	**0.014**	**−2.01**	**0.0448** *
**Education**	**−0.020**	**0.037**	**−0.53**	**0.593**
**Gender (female)**	**−0.665**	**0.207**	**−3.21**	**0.0013** **
Clock symmetry (success)	−0.950	0.240	−3.97	<0.0001 ***
Clock time (success)	−1.548	0.215	−7.20	<0.0001 ***
Verbal fluency (success)	−0.280	0.023	−12.26	<0.0001 ***
BMI	−0.044	0.022	−2.06	0.039 *

## Data Availability

Restrictions apply to the availability of this data. Data were obtained from the Alzheimer’s Disease Neuroimaging Initiative (ADNI) and are available at the official ADNI webpage, https://adni.loni.usc.edu/, accessed on 28 July 2023 with the permission of the ADNI Data and Publications Committee.
